# Fetal testis organ culture reproduces the dynamics of epigenetic reprogramming in rat gonocytes

**DOI:** 10.1186/s13072-017-0127-3

**Published:** 2017-04-11

**Authors:** Arlette Rwigemera, Fabien Joao, Geraldine Delbes

**Affiliations:** Institut National de la Recherche Scientifique, Centre INRS – Institut Armand-Frappier, 531, boulevard des Prairies, Laval, QC H7V 1B7 Canada

**Keywords:** Male germ cells, Gonocytes, Rat, Organ culture, Epigenetic reprogramming, 5mC, DNA methylation, Imprinted genes, Histone 3 modifications, H3K4me2, H3K4me3

## Abstract

**Background:**

Epigenetic reprogramming is a critical step in male germ cell development that occurs during perinatal life. It is characterized by the remodeling of different epigenetic marks such as DNA methylation (5mC) and methylation of histone H3. It has been suggested that endocrine disruptors can affect the male germline epigenome by altering epigenetic reprogramming, but the mechanisms involved are still unknown. We have previously used an organ culture system that maintains the development of the different fetal testis cell types, to evaluate the effects of various endocrine disruptors on gametogenesis and steroidogenesis in the rat. We hypothesize that this culture model can reproduce the epigenetic reprogramming in gonocytes. Our aim was to establish the kinetics of three epigenetic marks throughout perinatal development in rats in vivo and compare them after different culture times.

**Results:**

Using immunofluorescence, we showed that H3K4me2 transiently increased in gonocytes at 18.5 days post-coitum (dpc), while H3K4me3 displayed a stable increase in gonocytes from 18.5 dpc until after birth. 5mC progressively increased from 20.5 dpc until after birth. Using GFP-positive gonocytes purified from GCS-EGFP rats, we established the chronology of re-methylation of *H19* and *Snrpn* in rat gonocytes. Most importantly, using testis explanted at 16.5 or 18.5 dpc and cultured for 2–4 days, we demonstrated that the kinetics of changes in H3K4me2, H3K4me3, global DNA methylation and on parental imprints can generally be reproduced ex vivo with the model of organ culture without the addition of serum.

**Conclusions:**

This study reveals the chronology of three epigenetic marks (H3K4me2, H3K4me3 and 5mC) and the patterns of methylation of *H19* and *Snrpn* differentially methylated regions in rat gonocytes during perinatal development. Most importantly, our results suggest that the organ culture can reproduce the process of epigenetic reprogramming and can be used to study the impact of environmental chemicals on the establishment of the male germ cell epigenome.

**Electronic supplementary material:**

The online version of this article (doi:10.1186/s13072-017-0127-3) contains supplementary material, which is available to authorized users.

## Background

Germ cell development in mammals is a unique and complex process encompassing cell migration, proliferation, quiescence, differentiation and meiosis. Germ cell differentiation is initiated in the embryo when a small number of cells from the epiblast, the primordial germ cells (PGC), acquire the germ cell lineage fate and migrate to colonize the genital ridge [1]. These cells subsequently commit to male or female developmental pathway depending on the surrounding somatic environment. In the male gonad, Sertoli cells surround the germ cells, named gonocytes [2], forming seminiferous cords. Gonocytes proliferate before entering a quiescent phase where the cells are in G0 cell cycle arrest [3]. After birth, mitosis resumes and the gonocytes progressively differentiate into spermatogonia, which are responsible for the continuous sperm production in adulthood [3]. In brief, perinatal development represents a key developmental step for the germ line as male germ cell fate is programmed.

One of the key events that occur in germ cells during that window of development is epigenetic reprogramming: a resetting of the epigenome that is critical for the re-establishment of parental imprints, erasure of epimutations and generation of the transcriptional identity of the germ line [4]. This reprogramming occurs in part through DNA methylation and posttranslational modifications of histones which are interrelated [4]. Briefly, in PGC, there is a unique transient loss of DNA methylation associated with the increased methylation of lysine K27 of histone 3 (H3K27me3) [4–6]. This is followed in gonocytes by a progressive de novo establishment of DNA methylation patterns specific to male germ cells [7, 8]. This event is associated with the methylation of lysines 4 and 9 of histone 3 (H3K4me3 and H3K9me3) [4, 9]. Importantly, it has been shown that the dynamics of epigenetic reprogramming in germ cells is finely tuned by DNA methyltransferases (DNMTs), histone methyltransferases (HMTs) and histone demethylases (HDMs) [8]. Disruption of these processes could have an impact on the subsequent germ cells. In fact, DNA methylation plays an important role in successful spermatogenesis as germ cell-specific (GCS) knockout mice for *Dnmt3a* or *Dnmt3l* or mice mutated for *Dnmt3c* are infertile due to meiotic arrest [8, 10]. In addition, impairment of epigenetic reprogramming during perinatal development has been shown to permanently affect the sperm epigenome, which in turn could lead to adverse progeny outcome [11–13].

It was shown that in utero exposure to man-made chemicals that can mimic steroid hormones such as vinclozolin, bisphenol A, or di-(2-ethylhexyl) phthalate, can affect DNA methylation in mature sperm [14–16]. These compounds are called endocrine disruptors (ED) and in the last 60 years, there has been increasing concern about their impact on male reproductive health [17]. Indeed, correlating with the rising production of ED, an increased frequency of human male reproductive disorders has been observed worldwide, including testicular cancer, disorders of sex development, cryptorchidism, hypospadias and poor semen quality [18, 19]. These disorders may result from impaired testicular development specifically during perinatal life as epidemiological and experimental data have established that exposure to ED early in life can induce permanent defects in male fertility [19–22]. We have shown that there is a specific window of development when gametogenesis is most sensitive to estrogen-like compounds [20]. Nevertheless, there are still gaps in the literature regarding the mechanisms by which EDs affect fetal germ cells. It was shown that such compounds can affect gene expression in gonocytes [16] potentially affecting germline differentiation, but very little is known about whether and how they affect epigenetic reprogramming.

This lack of knowledge is in part due to a need of a robust study model. Studies in vivo allow assessment of ED effects on male reproductive tract disorders such as hypospadias or cryptorchidism [23] as well as long-term transgenerational effects [16], but they require the use of several animals as opposed to in vitro models. One powerful in vitro system that reproduces the in vivo kinetics of perinatal testicular development is the organ culture of rat fetal testes [24]. This technique allows the preservation of both tissue structure and the interaction between the different cell populations of the testis. As with any model, this technique has limitations. In particular, the maintenance of testicular development can only be achieved for a few days. Nonetheless, we and others have used this approach as a toxicological test to evaluate the direct effects of various ED on gametogenesis and steroidogenesis in rodent and human testes [20, 25, 26]. But, it is still unknown whether the epigenetic reprogramming that occurs in male germ cells at that time is faithfully reproduced in vitro.

We hypothesize that organ culture of rat fetal testes can reproduce the epigenetic reprogramming in gonocytes. To test this hypothesis, we quantified three key epigenetic marks after different culture times: DNA methylation (5mC), dimethylation and trimethylation of histone H3 on lysine 4 (H3K4me2, H3K4me3). These marks were chosen because it was shown that 5mC and H3K4me3 vary during development and reprogramming in vivo [5, 7, 9]; however, little is known about H3K4me2. Importantly, as the chronology of epigenetic reprogramming has mostly been established in mice, this study also quantified the dynamics of these key epigenetic marks in rat gonocytes throughout perinatal development.

## Methods

### Animals

Transgenic Sprague–Dawley rats expressing germ cell-specific GFP (GCS-EGFP) were generated and generously provided by Hammer [27]. The animal studies were conducted in accordance with the guidelines set out by the Canadian Council of Animal Care (CCAC) and as reviewed and approved by the Institutional Animal Care and Use Committee of the INRS (Protocol Nº: 1510-06). Females were caged with males for one night, and vaginal smears were done the following day to identify sperm-positive females. That day was counted as 0.5 day post-coitum (dpc). Pregnant rats were euthanized by CO_2_ asphyxiation and subsequent cervical dislocation. The fetuses were removed from the uterus, decapitated and placed on ice.

### In vivo and in vitro sample collection

**Fig. 1:**
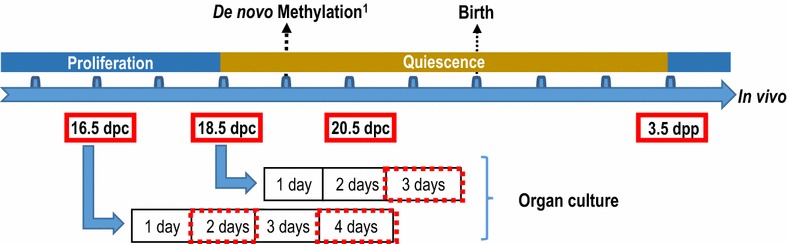
Schematic representation of our sampling design throughout gonocyte perinatal development in the rat. Rat testes were collected at four stages of development in vivo (*solid red boxes*) or at three time points after culture (*dashed red boxes*). For organ culture, testes were collected at 16.5 and 18.5 dpc and cultured for 4 and 3 days, respectively. ^1^Rose et al. [7]

To analyze expression levels of different epigenetic marks in vivo, testes were sampled at different stages of development covering the time of DNA re-methylation [7, 9]: 16.5, 18.5, 20.5 dpc and 3 days postpartum (dpp). For organ culture, testes were sampled at 16.5 or 18.5 dpc and maintained in culture for 3–4 days to reproduce the same developing time window as in vivo. Analyses were done on testes retrieved after 2 and 4 days of culture when explanted at 16.5 dpc, and after 3 days of culture when explanted at 18.5 dpc (Fig. 1). When sampled or at the end of the culture period, some testes were fixed while others were pooled for germ cell purification (Fig. 1).

### Organ culture

Testes were sampled from fetuses obtained at 16.5 and 18.5 dpc. Organ culture was done as previously described [24] on Millicell culture inserts (Millipore PICM01250, Etobicoke, Ontario, Canada) floating on culture media (DMEM/F-12, HEPES 15 mM), no phenol red; #11039-021, Life Technologies, Burlington, Ontario, Canada) containing 0.04 mg/mL Gentamicin (Life Technologies, #1570-060). Briefly, each explanted testis was placed in cold HBSS (Life Technologies, #14175095), cut in two or four equal parts when sampled at 16.5 or 18.5 dpc, respectively, and placed on a culture insert floating on 500 μL of media. Culture was maintained at 37 °C and 5% CO_2_ and media was changed daily.

### Immunofluorescence for H3K4me2 and H3K4me3

Testes sampled in vivo or after organ culture were immersed overnight in freshly prepared 4% paraformaldehyde fixative (Electron Microscopy Science, Hatfield, PA, USA), dehydrated, embedded in paraffin and cut in 5 μm sections for histological analysis. Because the GFP protein carried in germ cells by our transgenic animals could not be detected after fixation, we used HSP90 as a germ cell-specific marker [28]. Tissue sections were deparaffinized, rehydrated, submerged in the antigen retrieval tris buffer at pH 9 (tris 10 mM, EDTA 1 mM, tween 20 0.05%, pH 9) and microwaved for 3 min at full power or until the solution came to a boil then 7 min at 30% power. Slides were left to cool down in the solution for 15 min at room temperature and then rinsed with double-distilled water (ddH_2_O). All incubations with antibody/serum were done in a humidified chamber (Simport Scientific, Beloeil, Quebec, Canada) and subsequent washes were in PBS. Tissue sections were blocked with a 5% (*w/v*) BSA (Sigma-Aldrich, #A4503, Oakville, Ontario, Canada) solution for 30 min before incubation overnight at 4 °C with anti-H3K4me2 [29] (1:500, rabbit, #9726, Cell Signaling, Danvers, MA, USA) or anti-H3K4me3 [30] (1:500, rabbit, Cell Signaling, #9751) antibody in combination with the anti-HSP90 (1:200, mouse, #610419, BD Biosciences, San Jose, CA) diluted with 1% BSA in PBS. Slides were then washed and incubated with goat anti-mouse 488 (1:200, Life Technologies, #A11001) and goat anti-rabbit 594 (1:200, Life Technologies, #A11037) for an hour at room temperature. Following washes, slides were mounted in SlowFade Diamond Antifade Mountant with DAPI (Life Technologies, #S36972). Negative controls were done by omitting the primary antibodies. Moreover, non-cross-reactivity of the antibodies with unmodified histone H3 has been confirmed by Western blot (see Additional file 1: Figure S. 1).

### Immunofluorescence for 5mC

Following rehydration, tissue sections were incubated 5 min with pepsin (Sigma-Aldrich, #R2283) at 37 °C then washed with PBS before incubation with HCl 0.2 N for 10 min at 37 °C. Slides were washed and incubated 10 min with HCl 2 N at 37 °C. Following further washes, tissue sections were incubated with 5mC antibody [31, 32] (1:150, mouse, #A-1014-100, Epigentek, Farmingdale, NY, USA) diluted with 1% BSA in PBS overnight at 4 °C. Slides were then washed and incubated with goat anti-mouse 594 (1:200, Cell Signaling, #8890) and processed as above. Negative controls were done by omitting the primary antibody.

### Image capture and immunofluorescence quantification

Sections of all stages were placed on the same slide allowing comparison of immunofluorescence intensity. As a reference for normalization, one 16.5 dpc testis section from the same specimen was placed on each slide and used in all staining. Representative images of stained testicular sections were taken using a Nikon eclipse T*i*-S inverted microscope (Mississauga, Ontario, Canada) equipped with a DS-Ri2 camera and the NIS-Elements imaging software. Color pictures were first converted to monochrome using ImageJ software (National Institute of Health). The region of interest was determined as being inside the seminiferous tubules, and the Sertoli cell ring was distinguished from HSP90-immunostained gonocytes. Using the ImageJ ‘multi-point’ tool, four points were drawn in the nucleus of each gonocyte (HSP90-positive) and each Sertoli cell (HSP90-negative) of that region. This was repeated for 100 cells/section/biological replicate. The mean fluorescence (expressed in pixel intensity) was then calculated for each cell type and normalized using the value obtained for the reference 16.5 dpc testis sample.

### Germ cell purification

We used rats that express GFP (GFP+) specifically in germ cells which allows purification of germ cells at different stages of development or after culture. Explanted testes were pooled per litter and slightly cut to expose the seminiferous tubules, immerged in a solution of 1 mg/mL collagenase (Sigma, #C9891) and 0.02 mg/mL DNase (Sigma, #D4527) for 15 min at 37 °C and then centrifuged 5 min at 500*g*. The pellet was re-suspended in 0.25% trypsin–EDTA (Life Technologies, #25200-056) and incubated 10 min at 37 °C after which 10% fetal bovine serum (FBS) (Life Technologies, #10099-133) was added to neutralize trypsin. Cells were filtered with a 70 μm strainer (#352350, Fisher Scientific, Ottawa, Ontario, CA), centrifuged 5 min at 500 g and suspended in presort buffer (PBS 1X no Ca2+, no Mg2+ supplemented in EDTA 1 mM and HEPES 25 mM and 1% de FBS) prior to cell sorting with FACSJAZZ (BD Biosciences, San Jose, CA) at an event rate of ~2500/s. The sort gates were set on forward and side scatter, trigger pulse width and GFP expression. Cells were interrogated using a 488 nm laser, and emission was measured using 530/40 (FITC/GFP) and collected in FBS. After sorting, both GFP-positive and GFP-negative cell fractions were washed with HBSS and counted. Sorted cells were flash-frozen and stored at −80 °C until DNA extraction. After culture, 4–16 testes were pooled and processed similarly but without trypsin treatment. For each fraction at each stage, we evaluated the purity by calculating the percentage of GFP-positive or GFP-negative cells, respectively, on a hematocytometer (Table 1). GFP-negative fractions were all 100% pure. Importantly, GFP-positive cell fractions after culture showed low purity in terms of GFP signal but we noted that all exhibited typical gonocytes’ morphology (see Table 1; Additional file 1: Figure S. 2). This suggested that FACS sorting decreased the GFP signal in these cells.

**Table 1:** Purity of GFP-positive fractions obtained at sampling or after organ culture

Age of sampling	Percentage of GFP-positive cells	Percentage of gonocytes based on morphology
16.5 dpc	92.85 ± 1.13% (*n* = 13)	96.26 ± 1.09% (*n* = 13)
18.5 dpc	91.88 ± 1.18% (*n* = 4)	97.34 ± 1.55% (*n* = 4)
20.5 dpc	78.33 ± 2.91% (*n* = 4)	99.27 ± 0.73% (*n* = 4)
3 dpp	91.59 ± 2.78% (*n* = 5)	97.06 ± 2.24% (*n* = 5)
16.5 dpc + 2 d	66.00 ± 4.88% (*n* = 5)	97.71 ± 2.29% (*n* = 5)
16.5 dpc + 4 d	61.85 ± 9.04% (*n* = 4)	83.75 ± 13.89% (*n* = 4)
18.5 dpc + 3 d	61.27 ± 6.00% (*n* = 5)	97.01 ± 1.23% (*n* = 5)

Purity was evaluated on a hemocytometer based on fluorescence and morphology. All data are expressed as mean ± SEM (*n* = 4–13)

### DNA extraction

Cell pellets were thawed prior to extracting DNA using the Qiagen kit, QIAamp DNA micro (#56304, Qiagen, Toronto, Ontario Canada). Briefly, 30 μL of buffer ATL and 20 μL of proteinase K were added to cells and incubated for 1 h at 56 °C with pulse-vortexing every 30 min. After incubation, 50 μL of buffer ATL and 100 μL of buffer AL were added and mixed for 15 s. followed by the addition of 100 μL of filtered ethanol at 100%. DNA was then cleaned on columns as per manufacturer’s instructions and quantified using a spectrophotometer NanoDrop ND-1000 (Thermo Scientific, Wilmington, DE, USA). DNA was stored at −20 °C until use if not converted on the same day.

### Bisulfite conversion

Because the amount of DNA that was available for bisulfite conversion was limited by the number of sorted cells that could be obtained, especially after culture, we used different conversion kits depending on the amount of available DNA. The kit EpiTect Fast Bisulfite conversion (Qiagen, #59824) was used for samples with more than 500 ng of DNA while the EZ DNA Methylation-Direct (#D5020, Zymo Research, Irvine, CA, USA) was used for samples with less than 500 ng of DNA, with a minimum of 100 ng of DNA per reaction. Conversion was done as per manufacturer’s instructions, and converted DNA was stored at −20 °C until use.

### Pyrosequencing

The DNA methylation levels at differentially methylated regions (DMR) of paternally (*H19*) and maternally methylated (*Snrpn*) imprinted genes in both gonocytes and somatic cells were analyzed using the PyroMark Q24 Advanced (Qiagen). We used the CpG-rich repeat regions mapped by Stadnick et al. [33] to locate the studied DMR of *H19*. For *Snrpn*, genomic locations for the DMRs in the mouse established by Shemer et al. [34] were overlapped with the rat sequence (RGSC Rnor_6.0, July 2014) provided at http://genome.ucsc.edu. CpG-rich regions were then located using the CpG prediction program, MethPrimer [35]. The PyroMark Q24 Advanced software was used to design the best primers for the regions of interest: *H19* = forward: 5′-GGTTTTTAGGTGAT-TTGGGA-TATT-3′, biotinylated reverse: 5′-ACATTTAAATTTATAAAA-TAATCCCCTTCT-3′, sequencing: 5′-GTTGAATTTTAG-TTTTTTTTTATGG-3′. *Snrpn* = forward: TGGTGGTTT-GAGGTTGTTGAT, biotinylated reverse: 5′-TCCTAAAACCCAAAAACCATTCAATAAC-3′, sequencing: 5′-GGATGTAGGAG-TTATGT-3′. Assays were designed to analyze the methylation level of 7 and 5 CpGs for *H19* and *Snrpn,* respectively (Fig. 5a). Converted DNA was amplified using HotStarTaq kit (Qiagen, #203443). 50 to 100 ng of converted DNA was used per reaction in a volume of 25–50 μL with the following cycle repeated 50 times: 15 min at 95 °C, 15 s at 95 °C, 30 s at the annealing temperature (*H19* = 51 °C; *Snrpn* = 60 °C), 15 s at 72 °C; this was followed by a 10 min step at 72 °C. PCR products were stored at −20 °C until analysis. Amplified sequences were sequenced using the PyroMark Q24 Advanced CpG kit (Qiagen, #970922) and the PyroMark Q24 Vacuum Workstation as per manufacturer’s protocol.

### Statistical analysis

All values are mean ± SEM of 3 biological replicates obtained from different litters. All statistical analyses were done using GraphPad Prism 6 (GraphPad Software, La Jolla, CA, USA). The level of significance for all statistical tests was set at *p* < *0.05.* All parameters obtained in vivo were analyzed using a one-way ANOVA followed by Tukey’s test. The significance of the difference between the mean values of testes at the stage of sampling and testes after organ culture was evaluated using a one-way ANOVA followed by Tukey’s test at 16.5 dpc and using a Student’s unpaired *t* test at 18.5 dpc.

## Results

### Histone 3 methylation

**Fig. 2:**
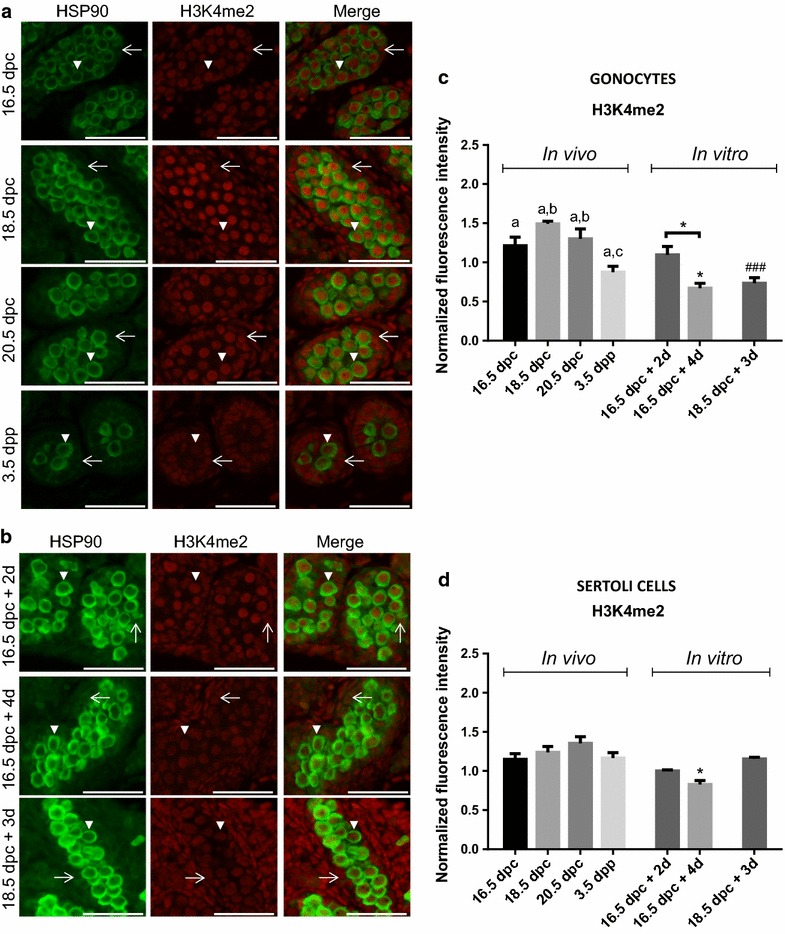
Global changes in H3K4me2 in gonocytes and Sertoli cells in vivo and in vitro. Co-staining of H3K4me2 (*red*) with HSP90 (*green*: marker of gonocyte) was done on sections of rat testes sampled in vivo (**a**) or after organ culture (**b**). Gonocytes (*arrow head*) and Sertoli cells (*arrow*) can be observed on sections of testis samples at different stages in vivo and in vitro. Immunofluorescence intensity was quantified in gonocytes (**c**) and in Sertoli cells (**d**). Data represent the average normalized fluorescence intensity ±SEM (*n* = 3/time point). ^a,b,c^ *p* < 0.05 between different stages in vivo using a one-way ANOVA followed by a Tukey’s post hoc test. *p < 0.05 between 16.5 dpc and different time of culture using a one-way ANOVA followed by a Tukey’s post hoc test. ^###^ *p* ≤ 0.001 between 18.5 dpc and after culture using a Student unpaired *t* test. *Scale* = 50 μm

**Fig. 3:**
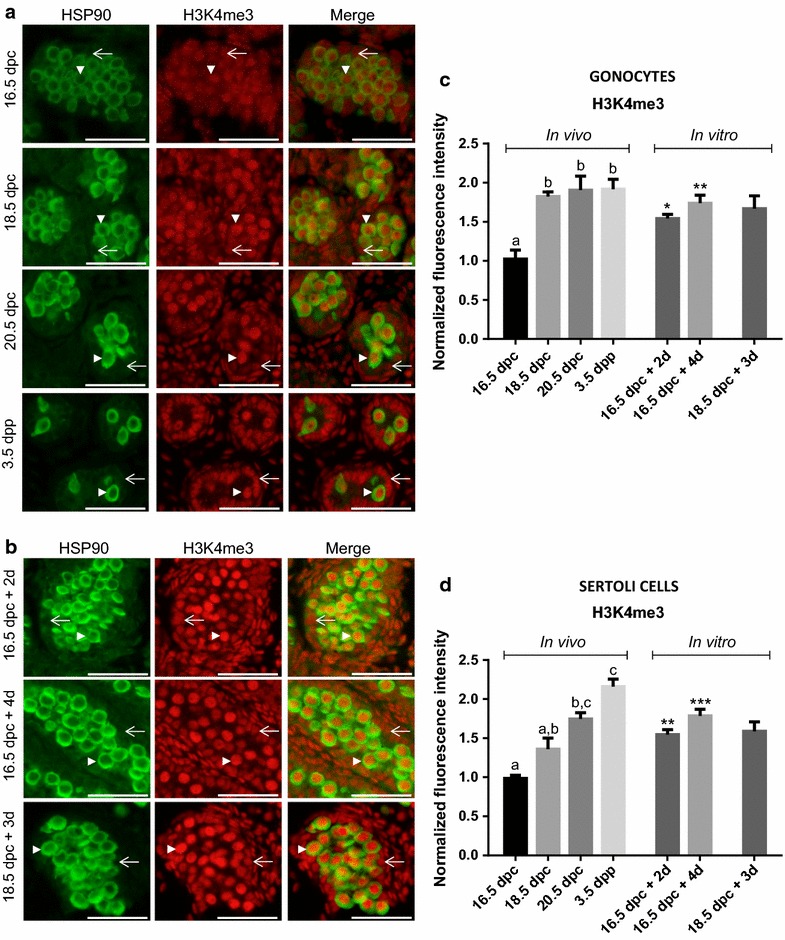
Global changes in H3K4me3 in gonocytes and Sertoli cells in vivo and in vitro. Co-staining of H3K4me3 (*red*) with HSP90 (*green*: marker of gonocyte) was done on sections of rat testes sampled in vivo (**a**) or after organ culture (**b**). Gonocytes (*arrow head*) and Sertoli cells (*arrow*) can be observed on sections of testis samples at different stages in vivo and in vitro. Immunofluorescence intensity was quantified in gonocytes (**c**) and in Sertoli cells (**d**). Data represent the average normalized fluorescence intensity ± SEM (*n* = 3/time point). ^a,b^ *p* < 0.05 between different stages in vivo using a one-way ANOVA followed by a Tukey’s post hoc test. *p < 0.05, **p ≤ 0.01, ***p ≤ 0.001 between 16.5 dpc and different times of culture using a one-way ANOVA followed by a Tukey’s post hoc test. *Scale* = 50 μm

We assessed the expression level of two histone 3 methylation marks known to vary during male mouse gonocytes’ development [4, 5, 9]. To test whether the in vitro kinetics followed the same pattern as in vivo in rat gonocytes, testes were sampled from different key stages of development, equivalent to different times of organ culture (Fig. 1). Using immunofluorescence co-staining, we assessed the expression of H3K4me2 (Fig. 2) and H3K4me3 (Fig. 3) in gonocytes (HSP90-positive cells) and Sertoli cells (HSP90-negative cells within the seminiferous cords).

#### Dimethylation of histone H3 on lysine 4 (H3K4me2)

H3K4me2 was present in the nucleus of all cell types in the fetal and postnatal testes at all stages studied, with an evident transient increase in gonocytes at 18.5 dpc (Fig. 2a). Further quantification confirmed such variation throughout perinatal development (Fig. 2c). Interestingly, H3K4me2 level in gonocytes was highest at 18.5 dpc and significantly decreased onwards until 3.5 dpp (Fig. 2a, c). In parallel, H3K4me2 levels were stable throughout time in Sertoli cells (Fig. 2d).

In vitro, the semi-quantitative study revealed a slight but significant decrease in H3K4me2 levels in Sertoli cells from testes cultured for 4 days when sampled at 16.5 dpc, a change that was not observed in vivo (Fig. 2b, d). In parallel, the level of H3K4me2 was not changed after 2 days of culture when testes were sampled at 16.5 dpc which suggest that the peak observed at 18.5 dpc in vivo was not reproduced in vitro. Interestingly, however, our data also showed that if the testes were sampled at 16.5 or 18.5 dpc, there was a significant decrease in the level of H3K4me2 in gonocytes in vitro at the end of the culture period (Fig. 2c). Overall, despite the shortcut of the peak observed in vivo, this decrease mirrored the significant decrease observed in vivo between 18.5 and 3.5 dpp, indicating that the kinetics of changes in H3K4me2 levels in gonocytes in vivo is partially reproduced in vitro.

#### Trimethylation of histone H3 on lysine 4 (H3K4me3)

H3K4me3 was also present in the nucleus of all cell types in the fetal and postnatal testes at all stages studied (Fig. 3a). Observations on tissue sections suggested an increase in the staining intensity with time especially in gonocytes (Fig. 3a). This was further confirmed by semi-quantitative analysis showing that H3K4me3 levels significantly increased between 16.5 and 18.5 dpc in gonocytes in vivo and remained stable until 3.5 dpp (Fig. 3c). Interestingly, a progressive increase in H3K4me3 level was also quantified in Sertoli cells with a significant difference at 20.5 dpc compared to 16.5 dpc (Fig. 3d).

In vitro, the semi-quantitative study demonstrated a significant increase in H3K4me3 levels in gonocytes and Sertoli cells after culture compared to the day of seeding (Fig. 3b–d). This increase was specifically observed in the testes taken at 16.5 dpc already after 2 days of culture and was then maintained. In addition, levels of H3K4me3 are maintained after 3 days of culture when testes were sampled at 18.5 dpc (Fig. 3b, d). These data demonstrate that the in vivo kinetics of changes in H3K4me3 levels in gonocytes and Sertoli cells are reproduced in vitro.

### DNA methylation (5mC)

#### Immunofluorescence

**Fig. 4:**
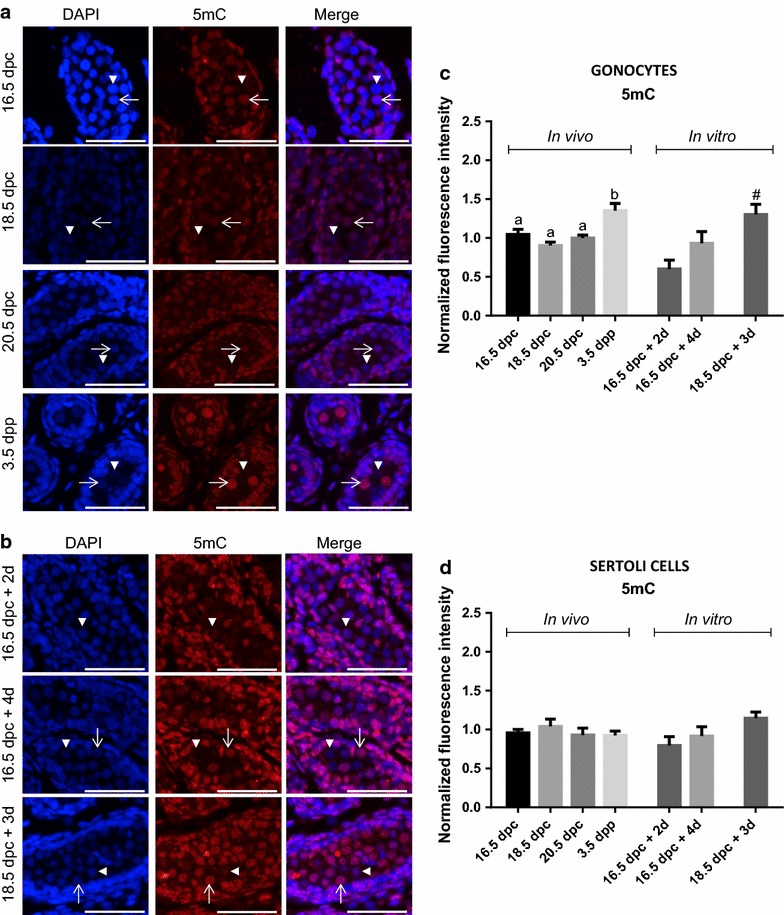
Global changes in DNA methylation in gonocytes and Sertoli cells in vivo and in vitro. Co-staining of 5mC (*red*) and DAPI (*blue*) was done on sections of rat testes sampled in vivo (**a**) or after organ culture (**b**). Note that at all age, we observed both unmethylated (*arrow head*) and methylated gonocytes (*arrow*). Immunofluorescence intensity was quantified in gonocytes (**c**) and in Sertoli cells (**d**). Data represent the average normalized fluorescence intensity ± SEM (*n* = 3/time point). ^a,b^ *p* < 0.05 between different stages in vivo using a one-way ANOVA followed by a Tukey’s post hoc test. ^#^ *p* < 0.05 between 18.5 dpc and after culture using a Student unpaired *t* test. *Scale* = 50 μm

Similar to what was done for histone 3 methylation, we evaluated global methylation of DNA in testicular cells using immunofluorescence for 5mC. Because co-staining with HSP90 was impossible due to antigen retrieval methods to reveal 5mC, we identified cells based on nucleus morphology. Indeed, gonocytes have round nucleus of about 10 μm diameter in the center of the seminiferous cords, whereas Sertoli cells have small lobular nucleus of about 4–5 μm diameter on the periphery of the cords [36]. We detected 5mC in the nucleus of all somatic cell types in the fetal and postnatal testes at all stages studied (Fig. 4a). On the other hand, only very low staining for 5mC could be observed in the nucleus of gonocytes from 16.5 to 20.5 dpc when the signal increased until 3.5 dpp (Fig. 4c). It is interesting to note that from 16.5 to 3.5 dpp, some gonocytes exhibit some staining (Fig. 4a, white arrow) while others did not (Fig. 4a, white arrowhead). Interestingly, when displaying data from individual cells, we observe a continuous variability of staining intensity at each stage of development suggesting that re-methylation does not occur synchronously in rat gonocytes (see Additional file 1: Figure S. 3). The semi-quantitative analysis of DNA methylation levels in Sertoli cells showed that it remained constant throughout the window of development studied (Fig. 4d).

In vitro, while constant staining could be observed in the nucleus of all somatic cells (Fig. 4b, d), we observed no or very low staining for 5mC in all gonocytes in testes sampled at 16.5 dpc after 2 days of culture (Fig. 4b). After 4 days of culture, some gonocytes became positive for 5mC (Fig. 4b, white arrow) while others remained negative (Fig. 4b, white arrowhead). This observation was confirmed by semi-quantitative analysis showing a decrease in 5mC staining in gonocytes from testes sampled at 16.5 dpc after 2 days of culture, compared to staining level at 16.5 dpc in vivo (Fig. 4c). Furthermore, although nonsignificant, a global increase was observed from 2 to 4 days of culture suggesting a re-gain of methylation that mirrors the in vivo kinetics (Fig. 4c). Interestingly, the level of 5mC significantly increased after 3 days of culture in gonocytes from testes sampled at 18.5 dpc (Fig. 4c), suggesting a re-methylation following the same kinetics as in vivo (Fig. 4b, c). In parallel, 5mC level in Sertoli cells in vitro did not vary at any time of culture studied (Fig. 4d) reproducing what was observed in vivo.

#### Pyrosequencing

**Fig. 5:**
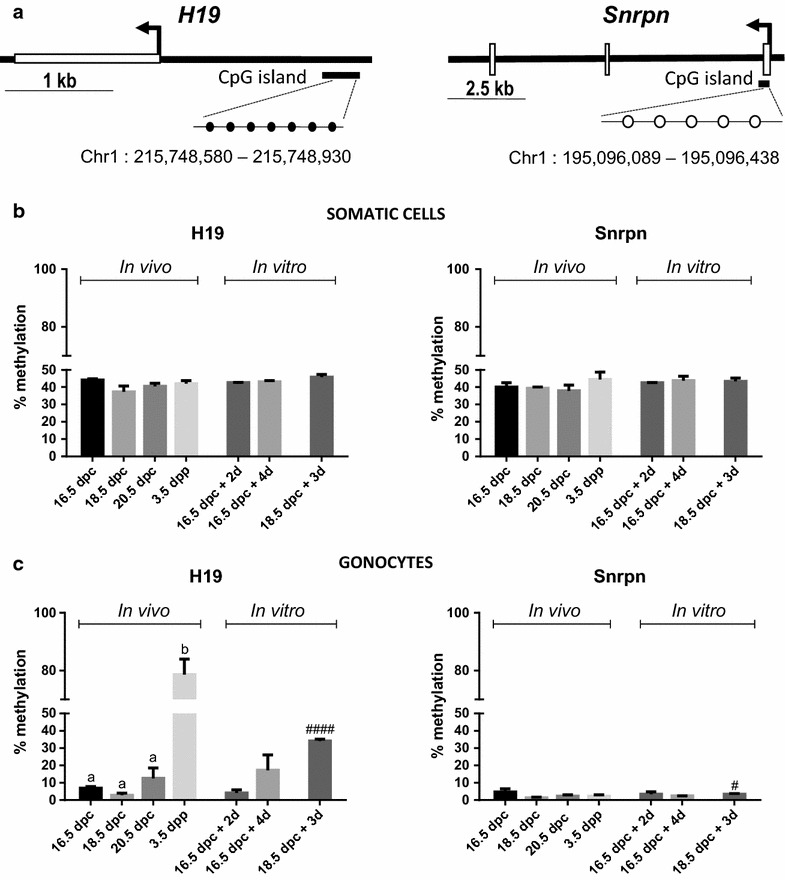
*H19* and *Snrpn* DMR methylation profiles in gonocytes and somatic cells in vivo and in vitro. **a** 7 CpG and 5 CpG sites were analyzed in *H19* (*black circles*) and *Snrpn* (*white circles*) DMR, respectively. The average percentage of methylation was obtained in GFP-positive gonocytes (**b**) and GFP-negative somatic cells (**c**) at different stages in vivo and in vitro. Data represent the average % methylation ± SEM (*n* = 3/time point). ^a,b^ *p* < 0.05 between different stages in vivo using a one-way ANOVA followed by a Tukey’s post hoc test. ^#^ *p* < 0.05, ^####^ *p* ≤ 0.0001 between 18.5 dpc and after culture using a Student unpaired *t* test

To test whether normal DNA methylation patterning occurred in our organ culture model following the same pattern as in vivo, we first established the kinetics of re-methylation of the DMRs of *H19* (paternally methylated imprinted gene) and *Snrpn* (maternally methylated imprinted gene) in rat male gonocytes. Using pyrosequencing on genomic DNA extracted from GFP-negative somatic cells, we showed that the DNA methylation levels of the *H19* and *Snrpn* DMRs ranged between 34.0 and 48.7% (Fig. 5b). A similar analysis on genomic DNA extracted from GFP-positive purified gonocytes revealed that *Snrpn* DMR methylation levels remained below 5% at all stages studied (Fig. 5c). Interestingly, the average *H19* DMR methylation level remained low in gonocytes between 16.5 dpc (6.86 ± 0.92%) and 18.5 dpc (2.86 ± 1.13%) but then increased from 20.5 dpc (12.57 ± 5.92%) until 3.5 dpp, where the highest level of methylation was measured (78.64 ± 5.33%; Fig. 5c). We also analyzed the level of DNA methylation in gonocytes at each CpG site for *H19* DMRs and observed that the increase in the average DNA methylation of *H19* DMR at 20.5 dpc was in fact due to two CpG sites (site #2 and #6) that stood out from the others (see Additional file 1: Figure S. 4). This indicated that those two sites are re-methylated earlier than the other five since their level of methylation also increase at 3.5 dpp.

In vitro, analysis of the DNA methylation of *H19* and *Snrpn* DMRs in GFP-negative somatic cells showed levels ranging from 41.2 to 47.3% which are similar to those measured in vivo. In addition, *Snrpn* DMR methylation levels in GFP-positive gonocytes did not vary in vitro over time when testes were sampled at 16.5 dpc but slightly increased after 3 days of culture of testes explanted at 18.5 dpc (Fig. 5c). On the other hand, in GFP-positive gonocytes purified after culture of testes sampled at 16.5 dpc, the level of methylation of *H19* DMR remained as low as the level at 16.5 dpc in vivo after 2 days but increased after 4 days of culture (Fig. 5c). Similarly, we observed that methylation levels of *H19* DMR significantly increased after 3 days of culture of testes explanted at 18.5 dpc (Fig. 5c). Looking at site-specific methylation level in gonocytes, it was interesting to note that sites #2 and #6 that were noted in vivo also stood out as the highest methylated CpG in vitro both at 16.5 dpc after 4 days of culture and at 18.5 dpc after 3 days of culture (see Additional file 1: Figure S. 4). These results show that the in vivo kinetics of *H19* and *Snrpn* DMRs methylation in male gonocytes is reproduced in vitro in rat fetal testis organ culture.

## Discussion

Epigenetic reprogramming is a key event occurring in germ cells during perinatal development and is essential for the establishment of germline fate. The dynamic changes in global DNA methylation and histone modifications associated with epigenetic reprogramming have mostly been described in the mouse [4, 9, 15]. In fact, it is generally considered that these global changes are conserved across mammalian species [37, 38], but the extent to which the exact timing is preserved remains largely unknown. In the present study, we have quantified three epigenetic marks that had been shown to vary in mouse gonocytes during perinatal development [4, 9, 15]. Our study is the first to characterize the in vivo dynamics of the histone modifications H3K4me2 and H3K4me3 in fetal male gonocytes of Sprague–Dawley rats during late gestation. Using immunofluorescence, we were able to quantify the pattern of H3K4me2 and H3K4me3 in gonocytes and Sertoli cells. We showed that H3K4me2 levels display a transient increase in rat gonocytes at 18.5 dpc and decreases thereafter until after birth. On the other hand, we showed that H3K4me3 was detectable in gonocytes at all stages studied and increased from 16.5 to 18.5 dpc to remain constant. While H3K4me2 had not been described in mice, the H3K4me3 pattern described in rats correlates nicely with what was observed in mouse gonocytes where it has been shown to increase between 15.5 and 17.5 dpc. Interestingly, we observed that H3K4me3 levels also increased in Sertoli cells which, to our knowledge, had not been described before. Since H3K4me2 and H3K4me3 are related to transcription activation [5], their dynamic could be associated with the setting-up of the transcriptional identity of the different testicular cell types [39–41].

The global DNA methylation erasure in PGCs followed by a re-methylation in germ cells in a sex-specific manner has been described in mouse, rat, and human [4, 7, 42], but the specific timing might differ between species. For example, while in mouse, the onset of DNA re-methylation occurs at some imprinted loci and at repetitive elements from 15.5 dpc, data from Rose et al. [7] suggest that this occurs later in Wistar rat gonocytes. Indeed, in rats, there is a 2-day difference in gonocyte development so that the equivalent of mouse 15.5 dpc would be 17.5 dpc in rat [43]. However, the onset of re-methylation in rat has been described at 19.5 dpc [7]. Our present data also support a delay in the re-methylation phases in rat gonocytes compared to mouse gonocytes as we characterized DNA re-methylation only from 20.5 dpc onward. This was observed both for global DNA methylation measured by immunofluorescence and, for the first time in rats, on two imprinted genes using pyrosequencing of genomic DNA obtained from purified gonocytes. Interestingly, our data also suggest that DNA re-methylation does not occur synchronously in rat gonocytes as from 16.5 to 3.5 dpp some we observed inter-nucleus variability (Fig. 4a, arrow and arrowhead). One could hypothesize that such changes are due to a change in the chromatin structure associated with cell cycle. But the fact that such variability can be seen at 20.5 dpc, a stage when all gonocytes are in G0 cell cycle arrest [43], suggests that it is not.

**Fig. 6:**
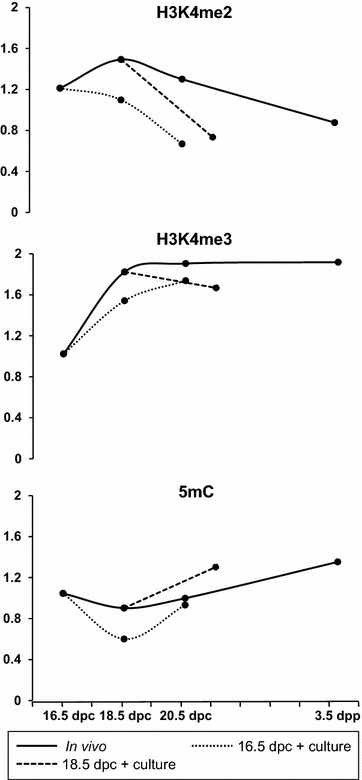
Summary of the dynamic changes in the levels of H3K4me2, H3K4me3 and 5mC in rat perinatal gonocytes in vivo (*full line*) and in vitro (*dashed line*)

It is known that DNA methylation and posttranslational modifications of histones are highly interrelated (reviewed in [44–46]). It was suggested that some histone modifications could be involved in the process of de novo methylation, but the mechanisms are still unclear [47, 48]. As some studies have shown that changes in H3K4me3 and H3K4me2 can precede or accompany de novo DNA methylation, these marks were proposed as good candidates to guide de novo DNA methylation [9, 47, 52]. On the other hand, others suggested that methylation of H3K4 may act as a protective marks from de novo methylation [48–51]. Such interaction in gonocytes has yet to be elucidated. Our findings show that in rat gonocytes, H3K4me2 levels decrease while H3K4me3 increases when 5mC increases (Fig. 6). Such kinetics of change suggests that histone 3 methylation may be involved in guiding de novo DNA methylation. Nevertheless, in the present study, only global changes were quantified which significantly limits the interpretation of such interaction on specific sequence. Further investigation using sequencing-based techniques would be required to elucidate such a dialogue.

Alterations in epigenetic reprogramming by EDs during perinatal life have been suggested to be at the origin of male fertility issues, affecting the DNA methylation pattern in mature sperm [15]. However, the mechanisms involved have not been elucidated yet. We have used the organ culture of rat fetal testes to study the impact of various ED on gametogenesis and steroidogenesis during perinatal life [20, 25, 26]. It was proved to be a robust model to reproduce the kinetics of development of the rat fetal testis [24]. In the present study, we have further characterized the in vitro development of gonocytes and Sertoli cells in this culture system, assessing the kinetics of changes in the three epigenetic marks studied in vivo. Our immunofluorescence quantification results show that the global kinetics of the three epigenetic marks studied were recovered in gonocytes maturing in vitro when compared to in vivo (Fig. 6). Indeed, DNA methylation patterns followed the same global increase as measured in vitro especially in testes explanted at 18.5 dpc after 3 days of culture. Similarly, the early increase and maintenance of H3K4me3 were faithfully reproduced. Only the H3K4me2 patterns were not completely faithful to those observed in vivo. Indeed, signal intensity for H3K4me2 was always lower in vitro than in vivo, in gonocytes and somatic cells, bypassing the peak observed in vivo at 18.5 dpc. This indicates that this specific mark may be more sensitive to culture conditions or that some exogenous factors influencing the pattern of that mark may be missing. Importantly, these results were obtained in media deprived of FBS and very similar data could be obtained when FBS was added to the culture media (see Additional file 1: Figure S. 5). This demonstrates that the epigenetic reprogramming occurs in fetal germ cells without the addition of any exogenous factor. The mechanisms guiding these changes in gonocyte chromatin are therefore driven only through autocrine or paracrine regulations.

Characterizing the methylation level of two imprinted genes in purified rat gonocytes before or after culture by pyrosequencing was made possible thanks to the use of GCS-EGFP rat testis [27]. The accuracy of the methylation levels measured by pyrosequencing largely depends on the purity of the fractions obtained. We have quantified that our GFP-positive fractions contained at least 83.75% of gonocyte-like cells and that the GFP-negative fractions contained 100% of cells negative for GFP signal. We believe our cell sorting method is valid first because in the GFP-negative fractions, we systematically obtained methylation levels that are very similar to the methylation profile expected in somatic cells. Moreover, the methylation level of *Snrpn* that is expected to be low in male germ cells [34] remained below 5% at all age studied in GFP-positive fractions. This confirms that our GFP-negative and GFP-positive fractions indeed represent somatic cells and gonocytes, respectively. Interestingly, our data show that the re-methylation of *H19* DMR that is expected in male gonocytes followed very similar patterns in vitro compared to the one described in vivo. Specifically, we have shown that re-methylation occurs first at specific CpG sites in vivo, a sequenced pattern that is reproduced in vitro. On the other hand, the demethylated status of the *Snrpn* DMR and the maintenance of DNA methylation in somatic cells were also demonstrated. To our knowledge, this is the first time that gonocytes have been purified from rat fetal testes maintained in culture in vitro, which offers great potential to study the molecular mechanisms by which epigenetics are regulated or altered specifically in these cells.

## Conclusions

Together the results of the present study describe the perinatal chronology of three epigenetic marks (H3K4me2, H3K4me3 and 5mC) and the pattern of methylation of *H19* and *Snrpn* DMR in rat gonocytes during perinatal development. Most importantly, we demonstrated that the chronology of those epigenetic marks can be reproduced in vitro with the model of organ culture without the addition of serum. Thus, we are confident that this model can be used to study the process of epigenetic reprogramming in rat fetal germ cells. Furthermore, this model represents a powerful tool to study the impact of environmental chemicals on the establishment of those epigenetic marks in fetal male germ cells.

## Additional file

**Additional file 1: Figure S. 1.** Validation of H3K4me2 and H3K4me3 antibodies cross-reactivity. Western blot was done with 10 μg of recombinant protein H3 (New England Biolabs, #M2503S, Whitby, Ontario, Canada). Membranes were incubated with anti-H3 (1/10,000) (A), anti-H3K4me2 (1/2000) (B) and anti-H3K4me3 (1/2000) (C). The expected band can be visualized with anti-H3 at ~15 kD, but no band could be detected using anti-H3K4me2 or anti-H3K4me3 demonstrating no cross-reactivity against the full unmodified histone H3. **Figure S. 2.** Representative  images of dispersed cells from rat fetal testis before and after FACS sorting. Testes were sampled at 18.5 dpc and cultured for 3 days prior to sorting by FACS. In the unsorted cell population (A), GFP-positive gonocytes (arrow) and GFP-negative somatic cells (arrow head) can be discriminated based on fluorescence but also difference in cell morphology. In the GFP-positive fraction (B), all cells exhibited gonocytes’ morphology when observed under bright field and some did not express GFP (*). Note that the GFP-negative gonocytes were stained with trypan blue (*), suggesting these cells died after sorting. Scale = 50 μm. **Figure S. 3.** Global changes in DNA methylation in gonocytes in vivo. Immunofluorescence intensity of 5mC was quantified in gonocytes (as in Fig. 4). Data are represented for 100 individual cells per testis at each time point (*n* = 3/time point). **Figure S. 4.** *H19* and *Snrpn* DMR methylation level per CpG site in gonocytes in vivo and in vitro. The percentage of methylation was obtained for each CpG site in GFP-positive gonocytes at different stages in vivo and in vitro. Data represent the average % methylation ± SEM (*n* = 3/time point). *: *p* < 0.05 between 16.5 dpc and different time points of culture using a one-way ANOVA followed by a Tukey’s post hoc test. #: *p* < 0.05 between 18.5 dpc and after culture using a Student unpaired *t* test. **Figure S. 5.** Summary of the dynamic changes in the levels of H3K4me2, H3K4me3 and 5mC, in rat perinatal gonocytes in vivo (full line) and in vitro with (red dashed line) or without FBS (black dashed line). Immunofluorescence intensity was quantified in gonocytes. Data represent the average normalized fluorescence intensity ± SEM (*n* = 3/time point). *: *p* < 0.05, **: *p* ≤ 0.01 between 16.5 dpc and different time points of culture using a one-way ANOVA followed by a Tukey’s post hoc test. #: *p* < 0.05, ###: *p* ≤ 0.001 between 18.5 dpc and after culture using a Student unpaired *t* test.

## Abbreviations

5mc: 5-methylcytosine
H3K4me2: H3 lysine 4 dimethylation
H3K4me3: H3 lysine 4 trimethylation
GFP: green fluorescent protein
HSP90: heat-shock protein 90
FACS: fluorescence-activated cell sorting
DMR: differentially methylated regions
