# Supplementary material for: Fetal testis organ culture reproduces the dynamics of epigenetic reprogramming in rat gonocytes
Source: Epigenetics Chromatin. 2017 Apr 11;10:19. doi: 10.1186/s13072-017-0127-3 (PMC5387332; doi:10.1186/s13072-017-0127-3)
Supplement: Supplementary file 1 — Additional file 1: Figure S. 1. Validation of H3K4me2 and H3K4me3 antibodies cross-reactivity. Western blot was done with 10 μg of recombinant protein H3 (New England Biolabs, #M2503S, Whitby, Ontario, Canada). Membranes were incubated with anti-H3 (1/10,000) (A), anti-H3K4me2 (1/2000) (B) and anti-H3K4me3 (1/2000) (C). The expected band can be visualized with anti-H3 at ~15 kD, but no band could be detected using anti-H3K4me2 or anti-H3K4me3 demonstrating no cross-reactivity against the full unmodified histone H3. Figure S. 2. Representative images of dispersed cells from rat fetal testis before and after FACS sorting. Testes were sampled at 18.5 dpc and cultured for 3 days prior to sorting by FACS. In the unsorted cell population (A), GFP-positive gonocytes (arrow) and GFP-negative somatic cells (arrow head) can be discriminated based on fluorescence but also difference in cell morphology. In the GFP-positive fraction (B), all cells exhibited gonocytes’ morphology when observed under bright field and some did not express GFP (*). Note that the GFP-negative gonocytes were stained with trypan blue (*), suggesting these cells died after sorting. Scale = 50 μm. Figure S. 3. Global changes in DNA methylation in gonocytes in vivo. Immunofluorescence intensity of 5mC was quantified in gonocytes (as in Fig. 4). Data are represented for 100 individual cells per testis at each time point (n = 3/time point). Figure S. 4. H19 and Snrpn DMR methylation level per CpG site in gonocytes in vivo and in vitro. The percentage of methylation was obtained for each CpG site in GFP-positive gonocytes at different stages in vivo and in vitro. Data represent the average % methylation ± SEM (n = 3/time point). *: p < 0.05 between 16.5 dpc and different time points of culture using a one-way ANOVA followed by a Tukey’s post hoc test. #: p < 0.05 between 18.5 dpc and after culture using a Student unpaired t test. Figure S. 5. Summary of the dynamic changes in the levels of H3K4me2, H3K4me3 a [file 13072_2017_127_MOESM1_ESM.pptx]

## Slide 1
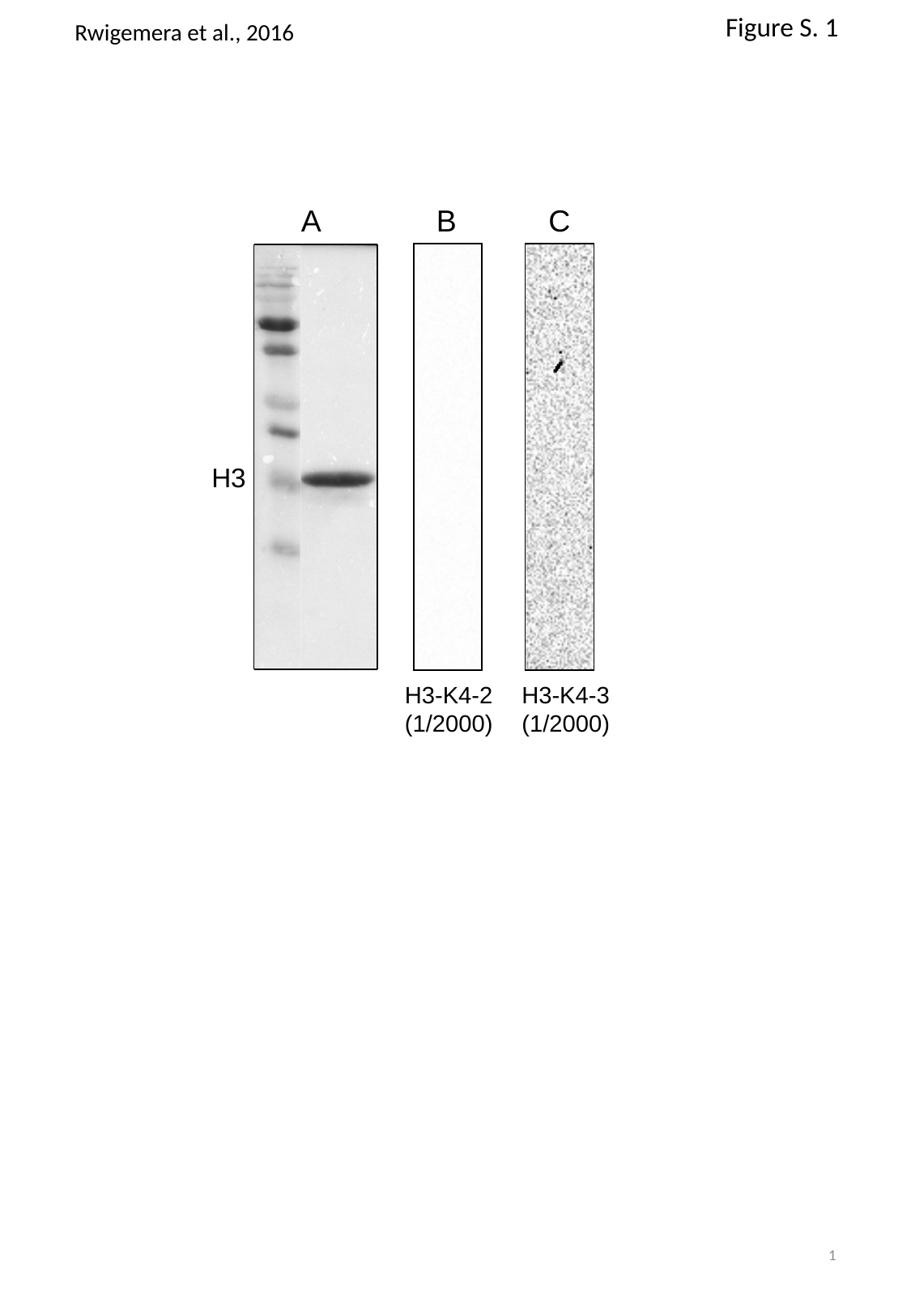

Figure S. 1
A
B
C
H3
H3-K4-2
(1/2000)
H3-K4-3
(1/2000)
1

## Slide 2
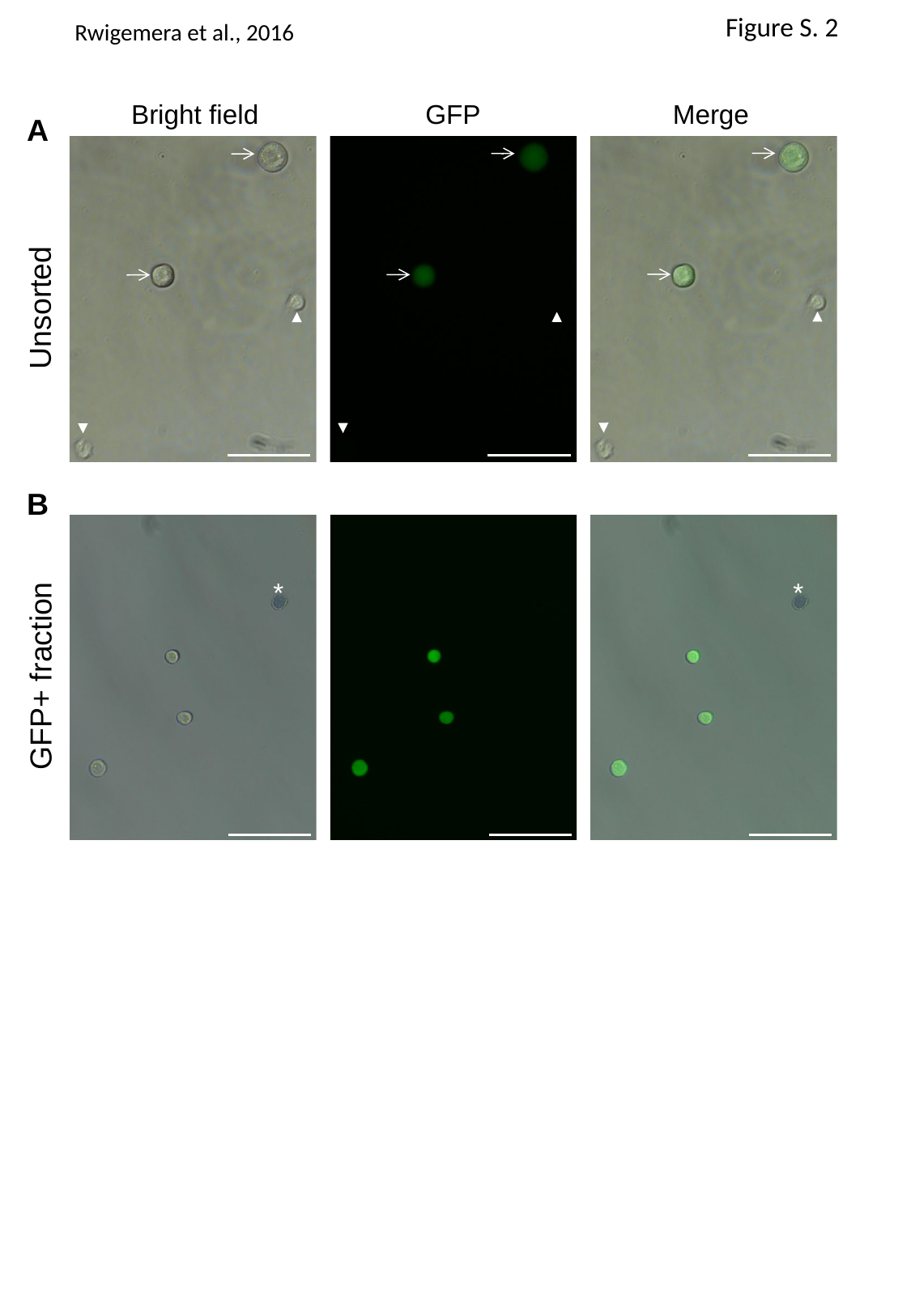

Figure S. 2
Bright field
GFP
Merge
A
Unsorted
B
*
*
GFP+ fraction

## Slide 3
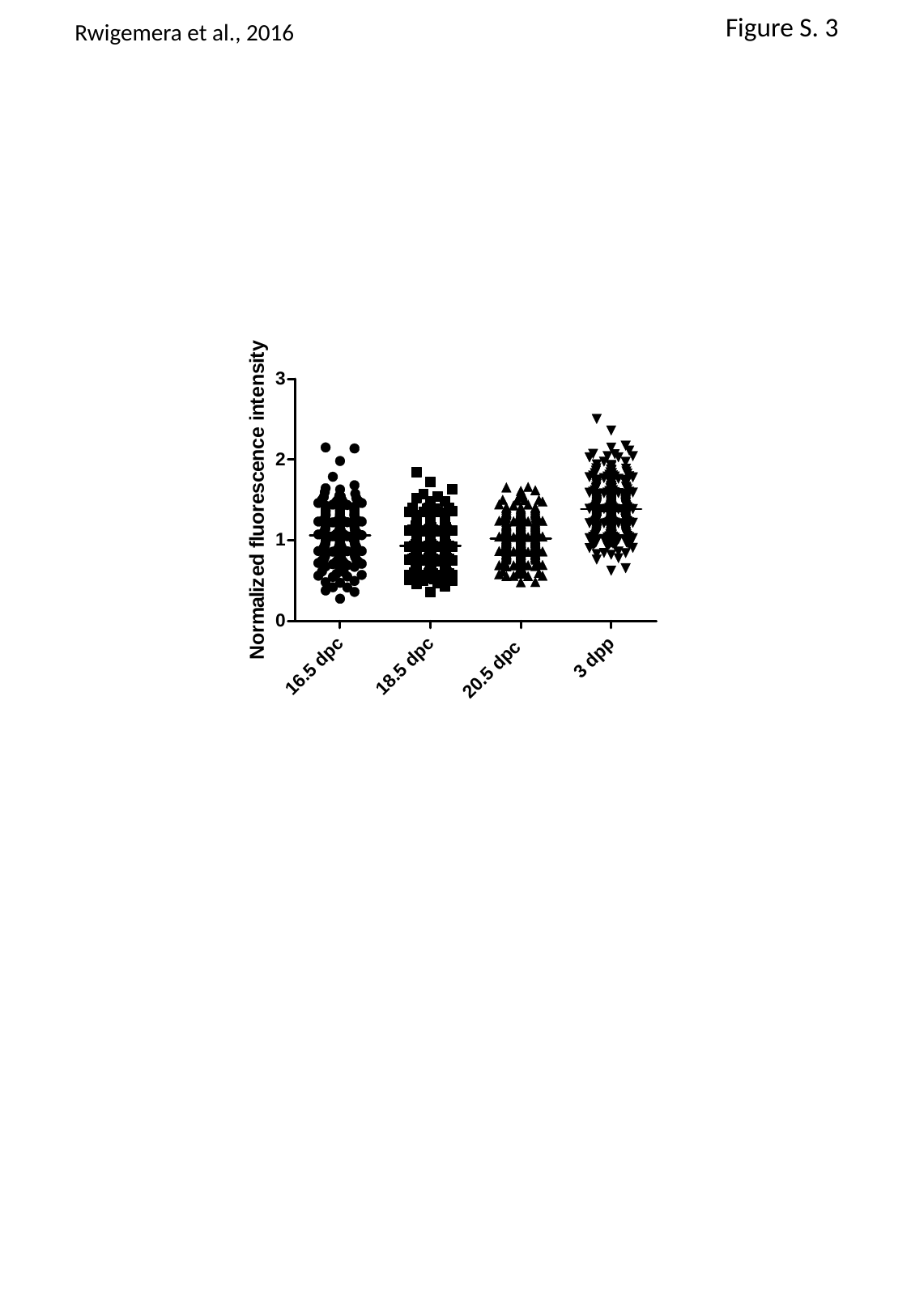

Figure S. 3

## Slide 4
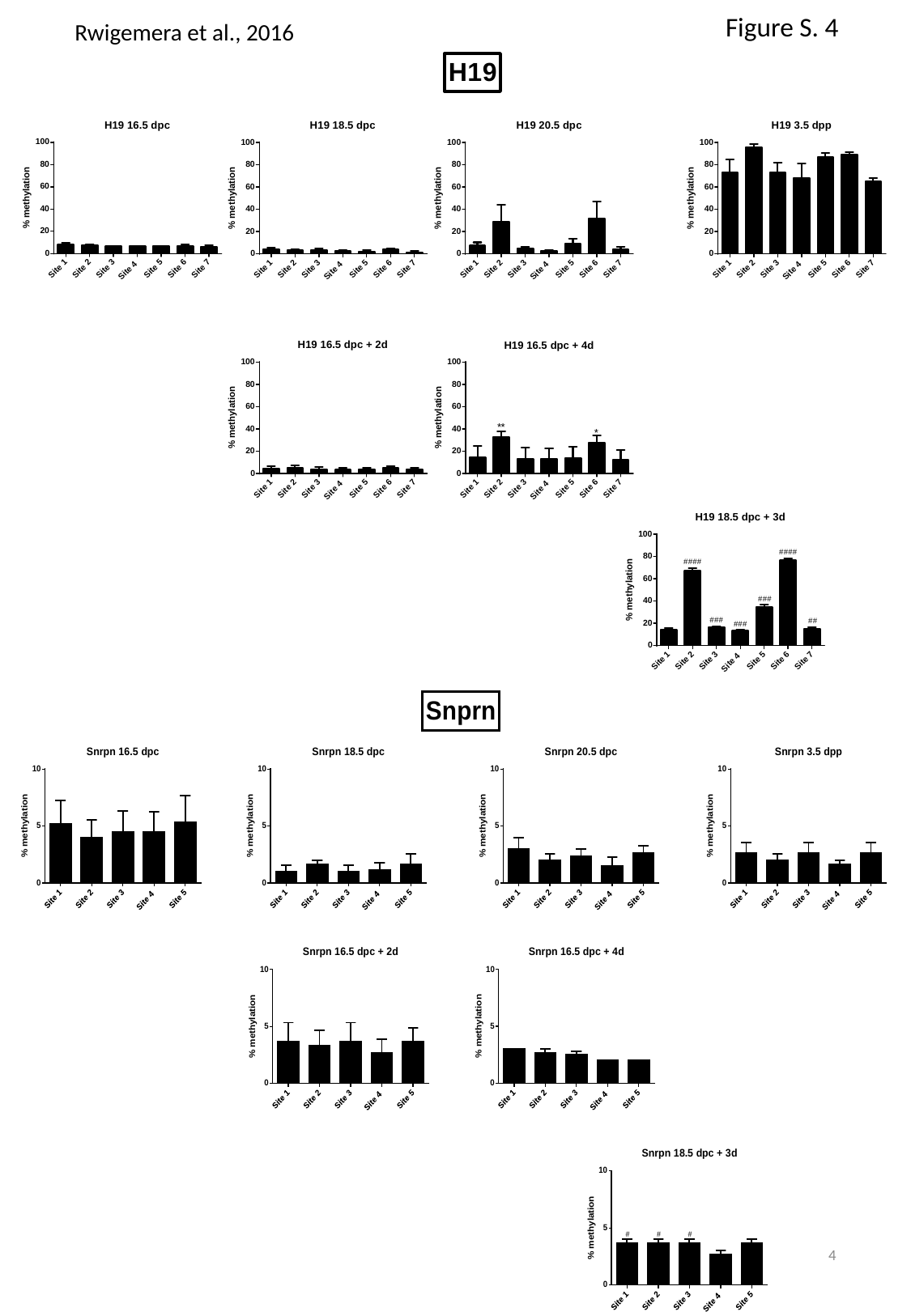

Figure S. 4
4

## Slide 5
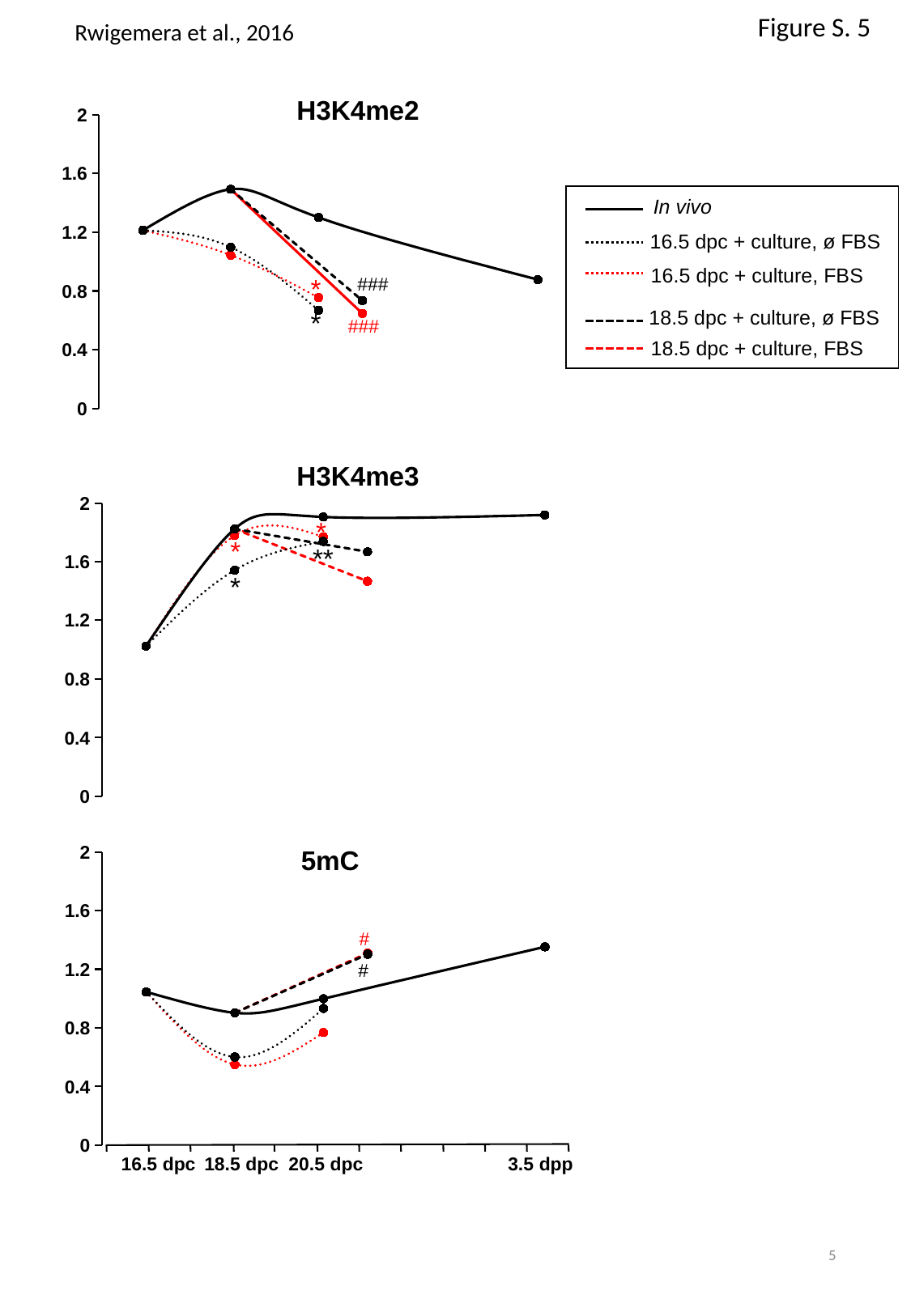

Figure S. 5
H3K4me2
### Chart
| Category | in vivo | in vitro | in vitro | in vitro | in vitro |
|---|---|---|---|---|---|
In vivo
16.5 dpc + culture, ø FBS
18.5 dpc + culture, ø FBS
16.5 dpc + culture, FBS
18.5 dpc + culture, FBS
###
*
*
###
H3K4me3
### Chart
| Category | in vivo | in vitro | in vitro | in vitro | in vitro |
|---|---|---|---|---|---|*
*
**
*
### Chart
| Category | in vivo | in vitro | in vitro | in vitro | in vitro |
|---|---|---|---|---|---|5mC
#
#
16.5 dpc
18.5 dpc
20.5 dpc
3.5 dpp
5
